# Parametric Study of Metallothermic Purification of Chloride Salts

**DOI:** 10.1021/acsomega.5c03893

**Published:** 2025-09-03

**Authors:** Adam Burak, Stephen Raiman

**Affiliations:** † 14736 University of Michigan, Nuclear Engineering and Radiological Sciences, 2355 Bonisteel Boulevard, Ann Arbor, Michigan 48109, United States; ‡ Arbor Halides, Suite 520, 333 Jackson Plaza, Ann Arbor, Michigan 48103, United States

## Abstract

A study was conducted to determine how variables during metallothermic purification of chloride salts affect the oxygen content of the final product. 100 g batches of ternary MgCl_2_–NaCl-KCl mixture (50–20–30 wt %) were purified for 3 h with flowing sparge gas and 0.25 wt % Mg addition as a reducing agent. After slag removal and remelting, oxygen content was measured via inert gas fusion analysis (IGFA). The as-received, melted, and baseline-purified salts had oxygen concentrations of 3466 ± 513, 889 ± 135, and 436 ± 151 ppm, respectively, with purification showing statistical significance (>99% confidence). A parametric study with 12 trials identified processing time as the most influential factor, as extending it to 6 h further reduced oxygen concentration to 143 ppm (>99% confidence). Variations in sparge and flush gas had no significant effect, and all tests used excess Mg. Uncertainty analysis of the IGFA technique estimated a 9% measurement uncertainty, primarily due to calibration standard variability.

## Graphical abstract

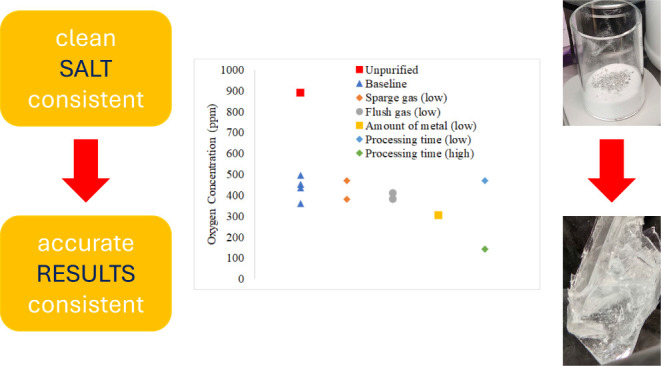

## Introduction

Molten salts make up a class of high-temperature liquids that can be beneficial for many applications. Their ability to be used at high temperatures without extreme pressure is part of what makes them attractive. This allows higher power conversion efficiencies^1^ and energy storage for technologies like molten salt reactors (MSRs), concentrated solar power (CSP), and thermal energy storage (TES). Unique chemistry also makes salts useful for things like actinide electrorefining, which is sometimes referred to as pyroprocessing. While the benefits of using salts are clear, there are also significant challenges. One of the most persistent problems in salt systems is corrosion. It is well-known that adding salt to water increases aqueous corrosion. Similarly, adding moisture to salt systems accelerates molten salt corrosion. Some commercial vendors can produce high purity salts, but this typically refers only to metals-basis impurities. Other impurities, like moisture or hydroxychlorides, are generally not quantified by vendors. Not only can increased oxygen and hydrogen based impurities (O/H impurities) in off-the-shelf salts accelerate corrosion, but they are generally not quantified by the vendor. This leaves it up to the researcher to purify the salt themselves. Otherwise they may experience serious damage to their equipment and/or inaccurate results. Raiman systematically explores this issue, finding that corrosion rates can be 10 times higher when using impure salt.^2,3^

There are a number of potential techniques for purifying salts, all with various trade-offs. This study focuses on chloride salts, which are an attractive candidate for MSRs and the next generation of CSP and TES because they remain stable at significantly higher temperatures than nitrate salts. Thermal dehydration, under an inert gas or vacuum, is a popular purification technique due to its ease of use. The drawback is that thermal dehydration may only be effective in converting salts to their monohydrate.^4,5^ Remaining moisture is expected to hydrolyze into corrosive species that persist once the salt becomes molten (eqs 1and 2).^6–10^ In addition, drying too fast may result in additional hydrolysis products from higher hydrates.

1 $$MCl+H_{2}O\to MOH+HCl$$

2 $$MCl+H_{2}O\to 2MO+HCl+\frac{1}{2}H_{2}$$

Several methods exist to dehydrate salts past the monohydrate or to remove impurities such as carbochlorination,^11^ electrochemical methods,^12–14^ and zone refining.^15^ The current work focuses on metallothermic purification (eq 3), using Mg as an active metal reducing agent. Recently explored in the literature,^16,17^ the benefits of metallothermic purification are that it is relatively simple to perform, it obviates the need for hazardous gas handling (if done properly), and the amount of resources required are relatively small.

3 $$Mg^{2+}+O^{2-}\to MgO$$

In this technique, a reductant is mixed with the solid salt and then heated to melting. The reductant reacts with oxygen bearing impurities in the salt, precipitating an insoluble product in the slag phase. After the process reaches an acceptable level of completion, it is solidified, and the slag phase is removed mechanically. The active metal reductant can theoretically be any metal that preferentially binds with the oxygen compared to the salt species. However, the only metal that has been tested in this work is Mg.

After the salt was purged, characterization is crucial to establish the composition of the salt. For experimental investigations, it is just as important as the purification itself. Characterization both proves that the purification was effective and quantifies the constituents of the salt. When purchasing salt from commercial chemical vendors, a specification sheet may be included. However, this typically specifies only metal-basis impurities and does not include moisture, which is a critical impurity for molten salt systems. The way that the salt is packaged and shipped may also contribute to the deterioration of the salt purity before it reaches the user. As metal-based characterization is well established, this work is focused on quantifying oxygen.

Several methods exist to quantify oxygen content in salts, such as loss-on-drying and Karl Fischer titration.^18,19^ However, these techniques are not effective for quantifying low concentrations of oxygen in the ppm range. Quantitative methods for determining oxygen concentrations are not well developed. Historically, experimenters working on the MSRE used the bromine method to quantify oxygen in the salt.^20^ However, the bromine technique was particularly complicated, requiring a custom apparatus was needed and highly competent analytical chemists. A well-funded, long-lived program enabled the development of the bromine method, but replicating those programmatic conditions in the current era may be challenging.

Recently inert gas fusion analysis (IGFA) has been under development to quantify oxygen in salts. This ex-situ technique uses a combustion analyzer to burn the sample in the presence of graphite and fluxing agents to liberate oxygen from some metal “M” as CO/CO_2_ (eq 4), depending on the machine and the reaction conditions.

4 MO+C→M+CO

Combustion products then flow through a reagent train designed to scrub out corrosive species like halides and species that may interfere with the measurement like moisture. In addition, CO may be oxidized to CO_2_ for measurement, depending on the machine. A combination of detectors, such as infrared (IR) and thermal conductivity, is used to quantify the combustion products. Combustion analysis is used extensively for measuring small concentrations of elements in steels, such as carbon, sulfur, and oxygen. There has been little work applying combustion analysis to salts, especially chloride salts. Though sparse, there is some recent work focused on adapting the IGFA technique to be used with salts.^21–23^ IGFA was also being developed for use at the same time as the MSRE.^24,25^ Issues with the technique include corrosion of instrument internals, developing a calibration strategy, and sample representativity.

The main purpose of this paper is to assess the effectiveness of processing parameters on metallothermic purification of the ternary chloride salt MgNaKCl (50–20–30 wt %), using Mg metal. The salt used was selected because purified MgNaKCl was required to support other projects within the lab. Mg was chosen as the active metal reducing agent, because there was precedent for it.^17,26^ Investigation of alternative reducing agents is outside the scope of this work. Parameters investigated for the study of the metallothermic purification process include: sparge gas flow rate, flush gas flow rate, amount of Mg added, and processing time. The effectiveness of the purification process will be assessed using IGFA to measure the oxygen content of the final product. As the IGFA technique is not well developed for salts, especially chloride salts, the characterization portion is a significant part of the work. The results from this work help to show what operational variables are most important during metallothermic purification of chloride salts and how to quantify the efficacy of the purification.

The previous work by Zhao is thorough and was performed well. They investigated the effects of changing the parameters on metallothermic purification. While the current work also parametrically investigated metallothermic purification, there are key differences. Zhao was developing a large-scale process, to be used on an industrial scale to reduce the MgOHCl concentration from weight percent levels to subweight percent levels. In Zhao’s study, industrial grade salts were used as a feedstock into the process, purifying a large-particle feedstock with around 1 wt % MgOHCl to around 0.1 wt % MgOHCl. The current study is focused on lab-scale processes to reduce the oxygen concentration in the salt. Feedstocks for the current study were 99% pure, or better, with an initial oxygen concentration in the 1000s of ppm and a goal of 100s of ppm oxygen. The fine powder of the current study’s feedstock required that the purification process be significantly modified. The main differences between the studies are the scale of the processes, the feedstock morphology, and the initial and final impurity levels.

## Experimental Section

### Experimental Materials

Salts were handled in an argon atmosphere glovebox (LC Technology Solutions), with atmosphere control resulting in <1 ppm of O_2_ and <1 ppm of H_2_O. A combustion analyzer was used to measure the concentration of oxygen in the samples (ELTRA OHp). Reagents and instruments in this project are listed in Table 1.

**Table 1:** Parts Used in These Tests Are Listed

Item	Supplier	Parameter	Part #
MgCl_2_	Thermo Scientific	99%	012315.A7
NaCl	Sigma-Aldrich	≥99.0%	S9888–5 kg
KCl	Thermo Scientific	99%	A11662.0I
Mg	Sigma-Aldrich	99.98%	254118–250G
Combustion Analyzer	ELTRA	N/A	OHp
Glovebox	LC Technology Solutions	Dry Ar atmosphere, 3-glove	N/A
ELTRA Crucible	ELTRA	N/A	90185
IGFA steel standard	ELTRA	110±11 ppm of O	91100–1007
Halogen trap Sb	ELTRA	N/A	90235
Halogen trap KI	ELTRA	N/A	90234
Tin capsules	ELTRA	5 mm size	90252
Shutze	ELTRA	N/A	90270
Mg perchlorate	ELTRA	N/A	90200
NaOH	ELTRA	N/A	90210
Balance (AND)	AND	210 g max	HM-202
Balance (Sartorius)	Sartorius	120 g max	Practum124–15
Quartz crucible (tapered)	MSE Pro	100 mL	JA1333
Quartz crucible (cylindrical)	MSE Pro	100 mL	JA1346
Quartz tubes	VWR	4 mm × 6 mm × 4’	75875–654
UHP Ar	Cryogenic Gases	UHP	AR.UHP-G580.300
UHP N_2_	Cryogenic Gases	UHP	NIT-50–300
Thermocouple	McMaster Carr	K-type	39095K64
Rotameter 1	Swagelok	0.01 SCFM	VAF-G1–05R–1–0-W
Rotameter 2	Swagelok	0.035 SCFM	VAF-G1–03R–1–0-W
Muffle furnace	Thermolyne	N/A	FB1415M
Dip rod	McMaster Carr	1/4”	89325K91
Sieve (large)	McMaster Carr	35 mesh (500 μm)	34735K525
Sieves (small)	McMaster Carr	140 mesh (106 μm)	34735K535

### Experimental Setup

A custom metallothermic purification setup was designed to perform these tests. The reaction mixture was contained in a tapered quartz crucible, and Ar gas was sparged through the salt using a quartz tube. A K type thermocouple was used to measure the salt temperature and was sheathed in a close-ended quartz tube. The thermocouple sheath was built in-house by melting and closing one end of a quartz tube. The same size quartz tubes were used for sparge tubes and thermocouple sheaths. Secondary containment consisted of a stainless steel boat emplaced under the crucible with enough volume to hold all of the salt. A gas containment system was designed to flush reaction products to an exhaust system outside the glovebox to prevent them from accumulating inside the glovebox. This consisted of a stainless steel cylinder, blinded with a plate on the top. The bottom was left open, resulting in partial containment, rather than a sealed system. The intent was to balance the convenience and capture efficiency of the volatile reactants. Four 1/4 in. (6.35 mm) compression fitting penetrations were welded to the top of the container for sparge gas supply, flush gas supply, thermocouple, and exhaust gas. UHP Ar was supplied via two rotameters to the sparge gas and flush gas ports. The exhaust gas was routed to a snorkel outside the glovebox. Gas handling entered the glovebox via a single feedthrough with three 1/4 in. (6.35 mm) stainless steel tubes. A muffle furnace was customized to heat the system. The heating element was removed, inverted, and reinstalled. A hole was drilled through the top to allow for top access. Loose ceramic fiber insulation was packed around the top of the container prior to heating. The temperature controller was rewired to control the temperature of the thermocouple in the salt. A diagram of the setup, sans furnace and secondary containment, is shown in Figure 1.

**Figure 1:**
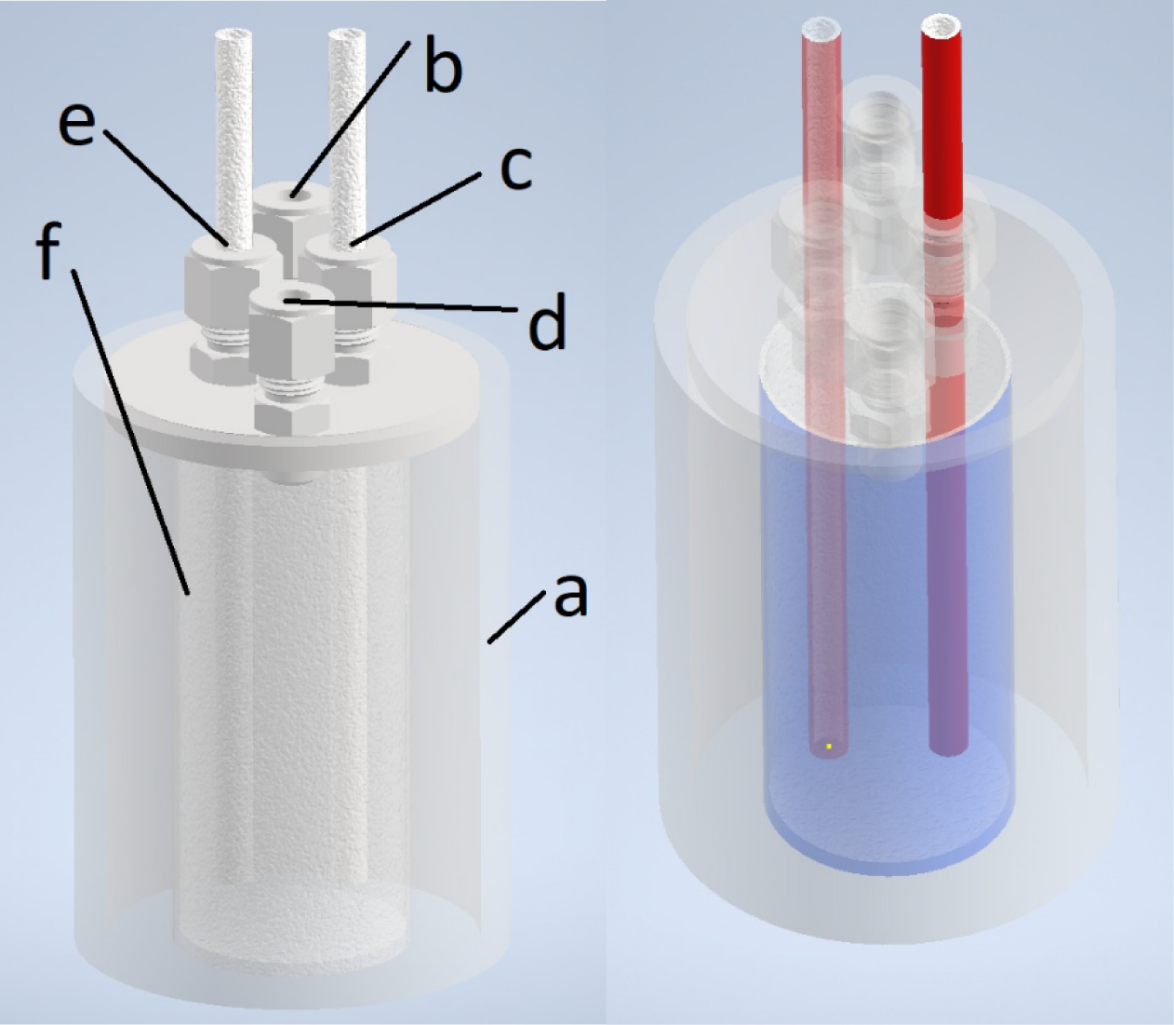
A diagram of the chloride salt purification setup is shown. On the left parts are detailed, including: (a) containment vessel, (b) flush gas inlet, (c) sparge gas inlet, (d) exhaust gas outlet, (e) thermocouple sheath, and (f) quartz crucible. On the right, the separate parts are shaded for better visibility.

### Metallothermic Purification

The salt used for these tests is a ternary mixture of salt, nominally MgCl_2_–NaCl–KCl (50–20–30 wt %). It was purified following a method similar to the one laid out by the National Renewable Energy Laboratory (NREL).^17^ MgCl_2_ was added to the crucible, followed by NaCl and then KCl. A spatula was used to stir the salt, roughly mixing it, and then Mg was added and pressed near the center of the salt with the spatula. Nominal additions are shown in Table 2. Test 00 did not purify the salt. It was melted and sampled to serve as an unpurified salt control.

**Table 2:** Nominal Salt aadditions for the the Parametric Study Are Listed

Test	Unit	MgCl_2_	NaCl	KCl	Mg
00	g	38.37	15.36	23.03	0
	wt %	49.99	20.01	30.00	0
01	g	51.07	20.12	30.13	0.28
	wt %	50.27	19.80	29.66	0.27
02	g	49.50	21.98	29.71	0.24
	wt %	48.80	21.67	29.29	0.24
03	g	50.08	20.04	30.44	0.23
	wt %	49.68	19.88	30.20	0.23
04	g	50.03	20.00	30.00	0.26
	wt %	49.88	19.94	29.91	0.26
05	g	50.00	20.02	30.00	0.26
	wt %	49.86	19.96	29.92	0.26
06	g	50.32	20.29	30.29	0.24
	wt %	49.75	20.06	29.95	0.24
07	g	50.14	20.05	30.02	0.29
	wt %	49.89	19.95	29.87	0.29
08	g	50.02	20.04	30.03	0.27
	wt %	49.84	19.97	29.92	0.27
09	g	50.05	20.02	30.03	0.11
	wt %	49.95	19.98	29.97	0.11
10	g	50.02	20.06	30.02	0.02
	wt %	49.96	20.03	29.98	0.02
11	g	50.03	20.08	30.02	0.24
	wt %	49.85	20.00	29.91	0.24
12	g	50.08	20.03	30.01	0.24
	wt %	49.90	19.96	29.90	0.24

The furnace was loaded with the secondary containment, then the crucible with salt, and then the containment was placed over the crucible from the top. Both quartz tubes, sparge gas, and thermocouple, were then inserted and worked down into the salt by pressing down and twisting. Insulation was packed on top of the gas containment to hold everything in place and mitigate heat loss. Gas lines were hooked up, flush gas was started, and the furnace was set to heat to 670 °C. Once the salt reached 670 °C, the sparge gas was turned on and the salt was held for some time. The isothermal holding time was chosen to be 3 h experimentally. At the end of the run both the quartz tubes were removed from the salt while it was molten, and the system was allowed to free cool overnight. Baseline conditions were used for tests 1–4. Subsequent tests modified parameters, as shown in Table 3.

**Table 3:** Experimental Conditions for the Parametric Study Are Listed^a^

Test	Sparge gas flow rate (SCFM)	Flush gas flow rate (SCFM)	Mass of Mg additive (g)	Processing time (hr)
00	**0**	**0**	**0**	**0**
01	0.01	0.035	0.27	3
02	0.01	0.035	0.24	3
03	0.01	0.035	0.23	3
04	0.01	0.035	0.26	3
05	**0.001**	0.035	0.26	3
06	**0**	0.035	0.24	3
07	0.01	**0.0035**	0.29	3
08	0.01	**0**	0.27	3
09	0.01	0.035	**0.15**	3
10	0.01	0.035	**0.05**	3
11	0.01	0.035	0.24	**1**
12	0.01	0.035	0.24	**6**

a Parameters that were modified from baseline are bolded.

After the salt was cooled the ingot was removed from the tapered crucibles. Any metallic Mg beads found on the top of the ingot (based on visible inspection) were chipped off with a chisel. The chisel was then placed around the bottom third of the ingot and struck with a hammer to remove the slag layer (Figure 2).

**Figure 2:**
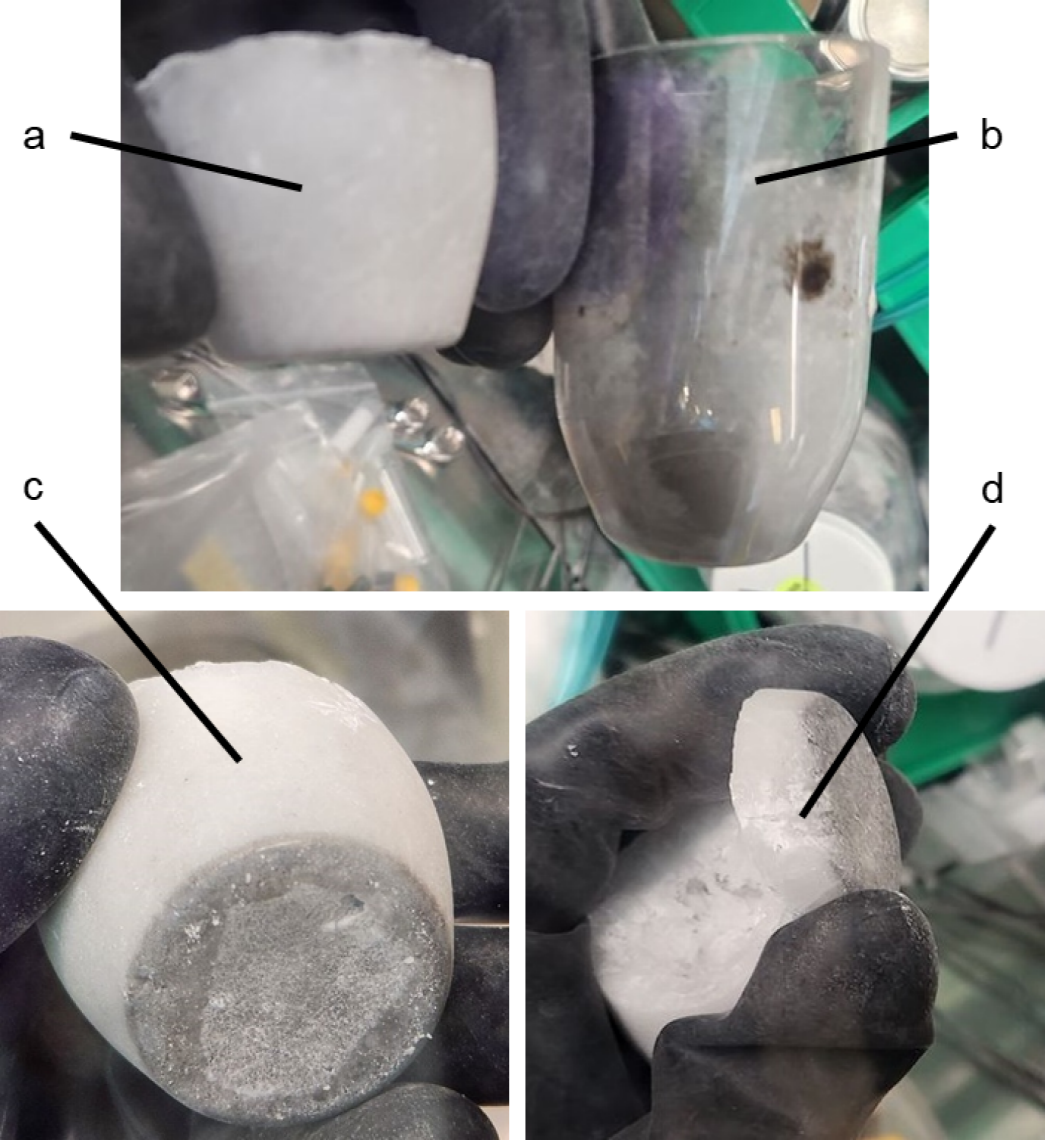
Purified salt is shown, including: (a) salt ingot, (b) used crucible, (c) salt ingot, and (d) separated slag layer.

After this process, the salt was placed in a clean cylindrical quartz crucible and melted to around 450 °C. A stainless steel rod was then used to collect approximately 20 g of sample via dip sampling. Dip samples are a reasonable compromise between convenience and representativity. Previous work shows that dip samples and liquid samples, which are assumed to be representative, resulted in similar oxygen concentration measurements when they were measured via titration. Dip samples were shown to be skewed, underestimating oxygen by around 8%.^27^ The collected dip samples were placed into a scintillation vial with a crushable lid, which was then sealed with electrical tape and stored in a glovebox until it was analyzed.

### Oxygen Concentration Measurements

To measure the concentration of oxygen in the salt samples, IGFA was used. The IGFA instrument, a combustion analyzer, features a graphite crucible, which makes contact between a lower electrode and an upper electrode to enable joule heating. The sample container consists of a reuseable outer graphite crucible and a single use inner graphite crucible. For salts, the inner graphite crucible commonly gets stuck in the outer graphite crucible. For this reason, single use graphite crucibles were used, which may be used in place of the two separate crucibles. For salt samples, a Sn flux was placed in the bottom of the graphite crucible. Once the actual run began, the crucible was outgassed, including powders and pellets added to it, by passing a relatively high power through the crucible for a short time. The power was then reduced to the reaction power and held constant until a stable background was obtained. Once the background was established, the sample was dropped into the molten bath. Oxygen in the sample binds with carbon from the crucible, or as an added powder, to produce CO/CO_2_. For this analysis, the outgassing power was 6000 W, and the reaction power was 4000 W.

After the sample reacts, the remainder of the IGFA technique varies depending on the instrument. Vendors may utilize different sensors or arrange their reagents/sensors in different ways. The two main sensors are nondispersive infrared detectors (NDIR) and thermal conductivity detectors. NDIR uses the absorption of light at specific wavelengths (4260 nm for CO_2_, 4670 nm for CO, 2700 nm for H_2_O, though other absorption bands may be used)^28,29^ to measure reactant gas concentrations. Thermal conductivity is as it sounds, measuring the change in thermal conductivity in response to changing gas concentration (18 mW/m*K for Ar, 187 mW/m*K for H_2_, 19 mW/m*K for H_2_O, 26 mW/m*K for N_2_, 25 mW/m*K for CO, 17 mW/m*K for CO_2_).^30^ The detectors used then dictate the reagents used to convert the gas. First, a filter is needed as a lot of dust is generated. Chemicals such as potassium iodide (KI) and antimony (Sb) are also used as part of the filter stage to remove corrosive gases, such as chlorine and fluorine, from the analysis stream to prevent damage to the machine. Other reagents may be used to scrub moisture and CO_2_, such as sodium hydroxide (NaOH) and magnesium perchlorate (Mg­(ClO_4_)_2_). Then other reagents, such as Schutze, can be used to oxidize H_2_ and CO to form H_2_O and CO_2_, respectively.

Each set of reagents may behave slightly differently, so a calibration must be performed after the reagents are changed or left to sit for some time. This also allows users to account for sources of bias (capsules, pellets, and crucibles). These calibration standards contain known concentrations of oxygen and include random uncertainty. Ideally, the calibration standards used should have concentrations similar to that of the sample. Calibrations were performed regularly throughout this work, with 110 ± 11 ppm oxygen steel pins. To calibrate the machine, 3 steel standards (weighed with Sartorius balance) and one blank were used. This calibration is in addition to the factory calibration, which does not change.

To prepare the samples, salt was weighed out in a porcelain mortar. The salt was crushed/ground briefly and then sieved between 500 and 106 μm. Any large particles that did not pass the top sieve were returned to the mortar and the process was repeated until the majority of the salt passed the top sieve. Salt on the 106 μm screen was then swirled around to ensure it was mixed and to allow fines to fall through. An AND high accuracy scale was used to tare a Sn capsule (approximately 200 mg), and then, it was filled with approximately 100 mg of salt. To seal the capsule it was folded once with the tweezers, folded again, pinched at the top with the tweezers, and then the corners were folded in (Figure 3). The sealed capsule was then weighed, and the salt mass was recorded. A marked sample tray was used to move Sn pouches filled with samples out of the glovebox. Triplicates were performed for each sample. If results were not consistent, additional samples were run until a pattern was discerned.

**Figure 3:**
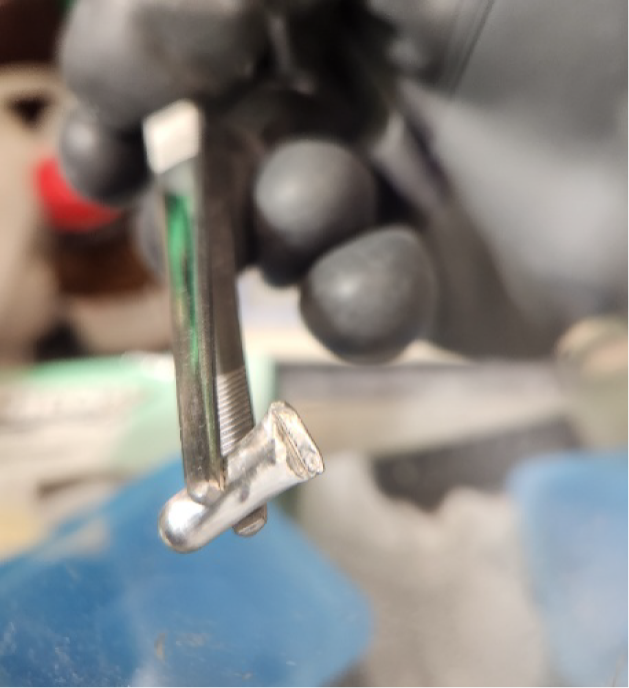
IGFA Sn sample pouch filled with salt was used for analysis.

### Statistical Significance

Several *t* tests were used to compare the averages in the oxygen concentration measured via IGFA between the various tests. Replicates were collected until there was a reasonable degree of confidence that the results were consistent. For the baseline purification, there was a sample size of 25. This was over 4 separate purifications with several IGFA replicates each. As the methodology developed, this number reduced to 3 for most tests. Degrees of freedom for each *t* test varied from 26 to 32. Table 4 lists the parameters used for the *t* tests.

**Table 4:** Parameters for T-Tests

Test	Test Conditions	IGFA Replicates	Average O (ppm)	St Dev O (ppm)
00	Unpurified	3	889	135
01	Baseline	6	360	151
02	Baseline	4	437	89
03	Baseline	8	495	151
04	Baseline	7	454	151
01–04	Baseline	25	442	152
05	Low sparge gas	9	382	99
06	No sparge gas	5	470	152
07	Low flush gas	5	382	140
08	No flush gas	3	411	101
09	0.15 wt % Mg	3	303	46
10	0.05 wt % Mg	3	304	55
11	1 h processing time	4	469	67
12	6 h processing time	3	143	50

## Results and Discussion

This project studies the metallothermic purification process of chloride salts developed at NREL,^17^ with some modifications. In the NREL process, a predrying step is used, in which the salt is heated to a lower temperature and held for several hours prior to heating to 670 °C. This step was omitted in the current study for two reasons. 1) The feedstock salt used in this work was much finer than the feedstock that Zhao used, 0.5 mm compared 3 mm, especially the MgCl_2_. The NaCl and KCl were similar to granulated sugar (around 500 μm), whereas MgCl_2_ was more like confectioner’s sugar (around 50 μm). Passing Ar through the fine salt at low temperature fluidized it and ejected much of the powder out of the crucible. 2) The feedstock salt used in this work was assumed to have a lower starting concentration of water because it was commercially anhydrous salt stored inside a controlled atmosphere glovebox. The predrying step Zhao used was designed to eliminate water from the system that was not chemically bound, as a result of storing the salt in ambient atmosphere. The feedstock salt used in this work had little water that was not chemically bound, which was confirmed in later tests. Other modifications were due to the constraints of the equipment that was used. Two rotameters were available for use in this work to control flow with ranges of 0.01 and 0.035 SCFM, which correspond to 4.7 and 16.5 cm^3^/s, respectively. The other constraint is the facility. A muffle furnace housed in a glovebox was used, which limits the size that can be processed to roughly 100 g.

Working within these constraints, an experimental matrix was designed to test 4 main parameters: sparge gas flow rate, flush gas flow rate, amount of reactive metal, and processing time. A set of 4 baseline tests were run with 4.7 cm^3^/s sparge gas, 16.5 cm^3^/s flush gas, 0.25 wt % Mg, and 3 h processing time. The parameters were then individually modified, as described in Table 3. After each purification run the salt ingot was removed from the crucible, and any slag or Mg inclusions were removed. The salt was then remelted in a clean crucible and dip samples were taken.

IGFA was used to quantify the amount of oxygen in the salts. For individual tests, triplicate measurements were taken. However, IGFA is a challenging technique that is still in its early development for chloride salts. As such, there was a significant amount of anomalous behavior during testing, such as machine errors. In addition, it was sometimes unclear whether the results were representative due to the high uncertainty of the technique. When necessary, as perceived by the machine operator (the lead author), additional test points were collected to clarify the data. In addition, all 4 tests performed with the baseline parameters (Salt_01 through Salt_04) were agglomerated into one data set. These resulted in different sample sizes for different tests. The data is displayed in the Table 5.

**Table 5:** Results from IGFA Tests

Test	Sample	Mass (mg)	Oxygen (ppm)	Average	St. Dev	Area
Salt_00	2	121	1080	889	135	86
	3	134	788			69
	4	141	800			74
Salt_01	2	224	237	360	151	237
	3	213	240			240
	4	190	320			320
	6	105	530			530
	7	70	605			605
	8	108	230			230
Salt_02	2	200	288	437	99	288
	3	167	566			566
	10	133	435			435
	12	89	458			458
Salt_03	3	156	695	495	151	695
	4	162	623			623
	5	141	607			607
	7	139	278			278
	9	105	349			349
	10	92	352			352
	11	113	633			633
	12	107	422			422
Salt_04	5	154	366	454	151	366
	6	119	478			478
	7	107	677			677
	8	103	670			670
	9	110	274			274
	11	122	322			322
	12	78	389			389
**Salt_01 through Salt_04 (baseline tests)**				**442**	**152**	
Salt_05	2	124	291	382	99	291
	3	106	422			422
	4	153	415			415
	4	109	327			327
	5	144	259			259
	6	137	409			409
	7	106	540			540
	9	97	516			516
	10	135	259			259
Salt_06	3	131	327	470	152	327
	4	156	533			533
	5	133	252			252
	6	109	627			627
	12	118	610			610
Salt_07	2	136	654	382	140	654
	3	132	369			369
	4	168	328			328
	5	118	282			282
	6	100	277			277
Salt_08	2	144	341	411	101	341
	3	117	338			338
	4	129	554			554
Salt_09	2	100	367	303	46	367
	3	134	282			282
	4	147	261			261
Salt_10	2	135	226	304	55	226
	3	141	351			351
	4	116	334			334
Salt_11	2	191	354	469	67	354
	5	129	513			513
	6	147	492			492
	7	162	518			518
Salt_12	2	141	97	143	50	97
	3	109	119			119
	4	128	213			213

In order to better convey the data, results are displayed using “violin” plots. A violin plot has similar information to those of a box and whisker plot. In the plots in this work, the white square is the mean of the data, white stars are interquartile ranges, and black diamonds are individual data points. Violin plots add one crucial element of information, insight into the probability density function. The width of the violin corresponds to the probability density at that point, similar to a histogram displayed vertically. Though the number of samples is relatively small, this may be useful to give an idea of how the points are grouped together with the high uncertainty IGFA results. Regardless, the individual points are displayed in the plots.

The individual constituents of the as-received salt were analyzed for oxygen content to get an idea of the starting concentration (Figure 4).

**Figure 4:**
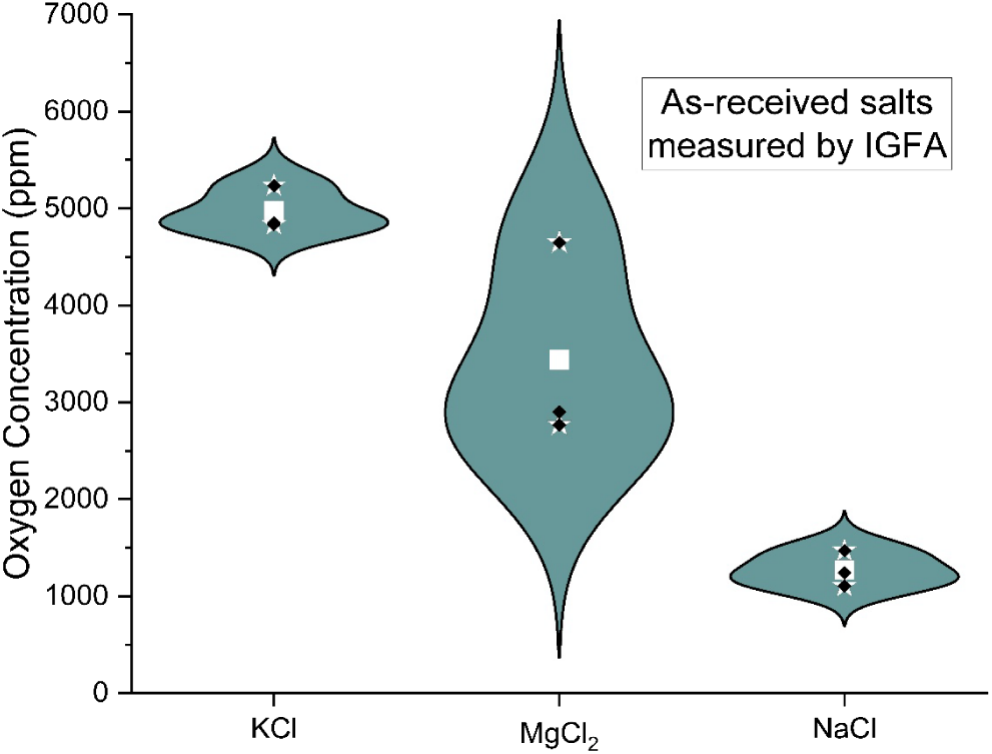
Oxygen concentration of as-received salts, as measured by IGFA.

The KCl had a relatively high amount of oxygen, an average of 4974 ± 184 ppm. Less oxygen was measured in MgCl_2_, an average of 3439 ± 855 ppm, but the uncertainty is significant. The NaCl showed the least oxygen with an average of 1273 ± 151 ppm. All three salts averaged together had and oxygen concentration of 3466 ± 513 ppm. The salt constituents were then mixed, melted, and then dip sampled to compare them to the baseline purification tests (Figure 5).

**Figure 5:**
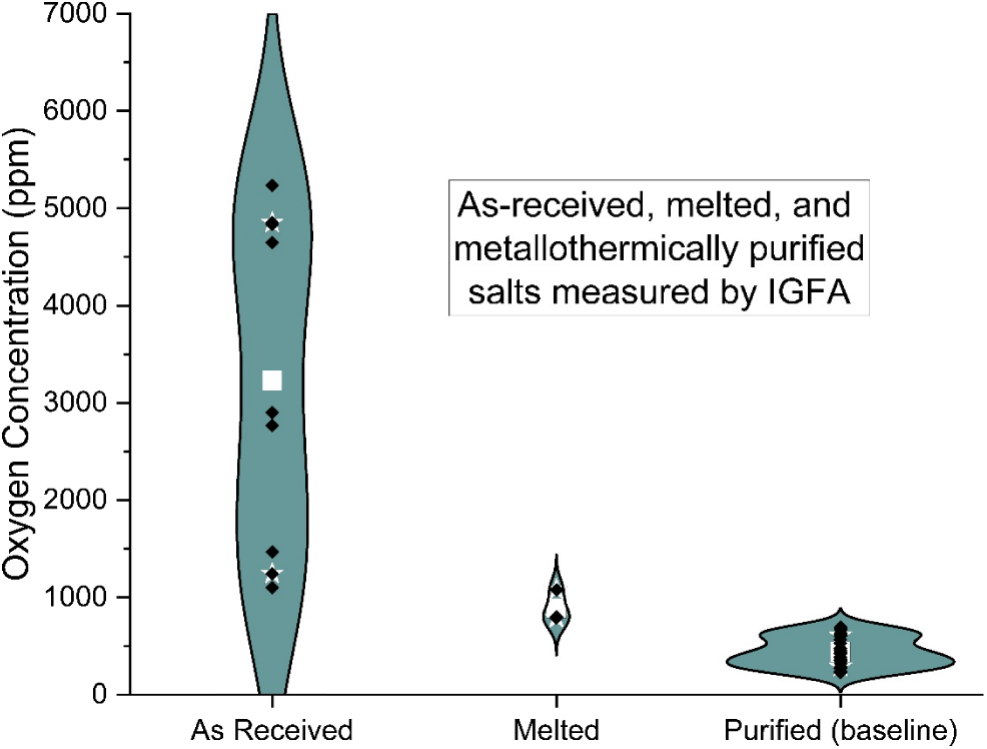
Oxygen concentration for as-received salts, melted salt, and baseline purified salt.

The melting process removed a significant proportion of oxygen from the salt, resulting in an average of 889 ± 135 ppm. Metallothermic purification of the salt removed even more O, bringing the average to 436 ± 151 ppm. After it was established that the purification process effectively and consistently reduced the oxygen content, a battery of tests was performed to determine which parameters were the most influential. The influence of sparge gas (the UHP Ar flowing through the salt) and flush gas (the UHP Ar sweeping the headspace) flow rates are shown in Figures 6 and 7.

**Figure 6:**
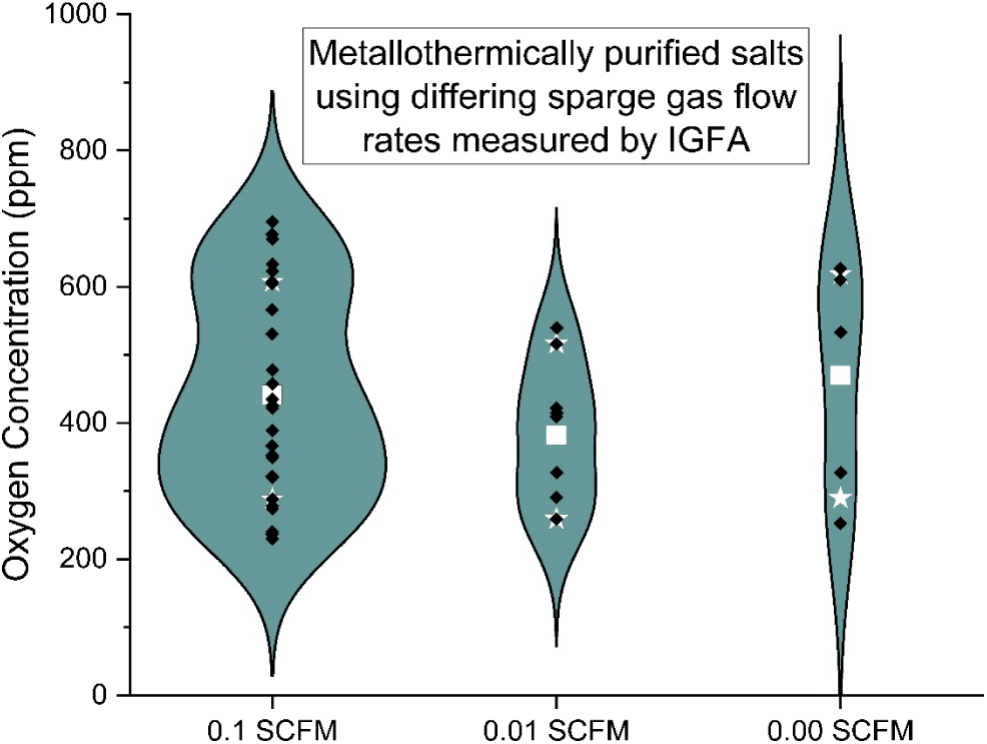
Oxygen concentration for metallothermically purified salt with varying sparge gas flow rates.

**Figure 7:**
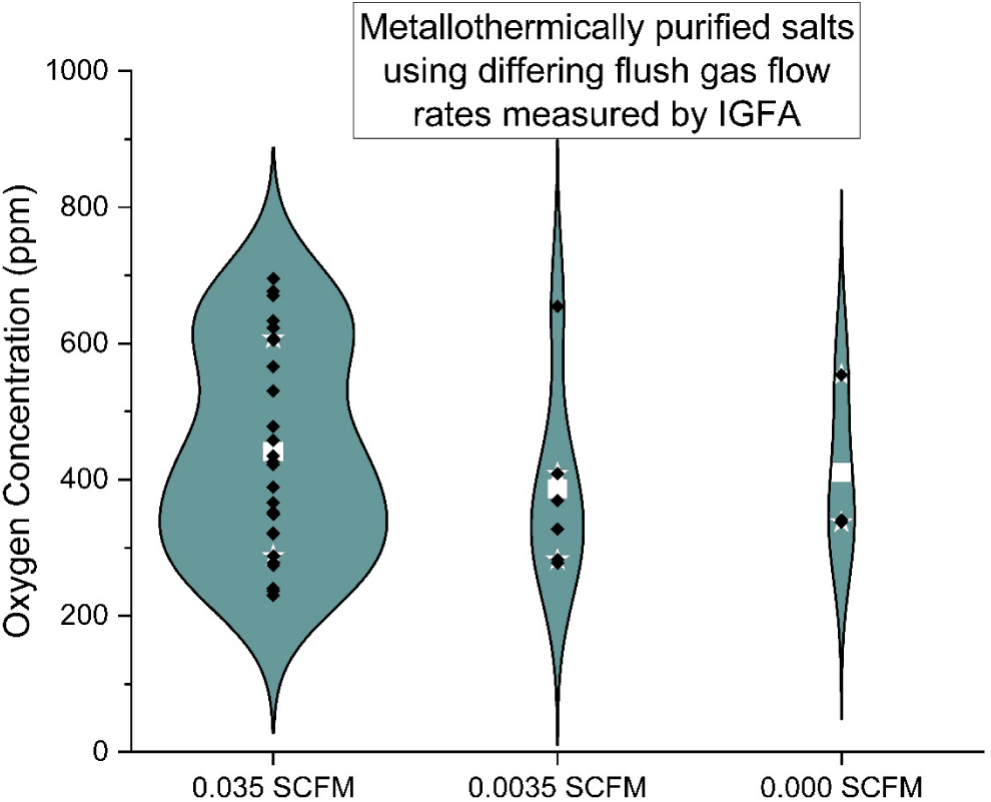
Oxygen concentration for metallothermically purified salts with varying flush gas flow rates.

The baseline gas flow rates used were 4.7 and 16.5 cm^3^/s for the sparge and flush gas, respectively. They were tested at the baseline flow, 10% flow, and no flow. A statistically significant change in oxygen concentration was not observed when the gas flow rates were changed. The main purpose of the sparge gas may be to mix the salt. Zhao also states “Sparging during both pre-drying and purification can facilitate fast evolution of H_2_O and HCl, which is essential to preventing back-reactions, reducing the time needed for both processes, and improving the overall salt processing rate and cost-effectiveness”.^17^ This suggests that sparging to mix the salt and processing time are interrelated and must be balanced as part of the process flowsheet. In a small system such as ours the effects of mixing to liberate reaction products may be limited but will likely become important in a larger purification system. For the flush gas, the main purpose is safety. The goal is to reduce the concentration of H_2_ in the effluent to safer levels, rather than some Le Chatelier type chemical effect (removing products to increase the reaction rate). Therefore, these results are unsurprising.

The results of varying the amount of Mg metal added are shown in Figure 8. The purpose of the Mg is to bind with oxygen containing impurities and precipitate out of the solution (eq 1). The baseline amount of Mg metal added was 0.25 wt %. This was to replicate the NREL process, which they chose by calculating the stoichiometric amount of Mg required. For this work, the concentration of Mg additive was tested at reduced concentrations of 0.15 and 0.05 wt %.

**Figure 8:**
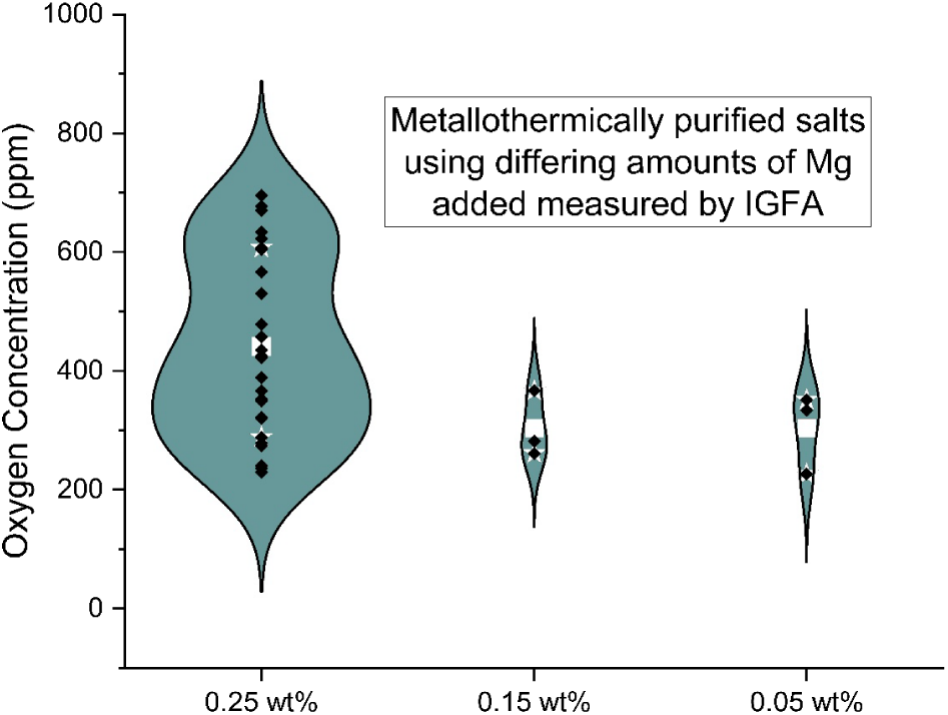
Oxygen concentration for metallothermically purified salt with varying amounts of Mg metal addition.

A slight decrease in the amount of oxygen in the salt treated with less Mg can be seen, but the effect was not statistically significant and is assumed to be experimental variability. the amount of Mg added had little effect on the amount of oxygen in the purified salt. Though counterintuitive, this was expected as the purity of the feedstock salt used in this work was higher than Zhao’s. Around 3500 ppm oxygen was measured in the unpurified salt in this work, compared to >1 wt % MgOHCl for NREL’s unpurified salt. At the beginning of the current tests the concentration of oxygen in the feedstock salt was unknown, so the stoichiometric amount of Mg required to purify the salt could not be calculated. The IGFA measurements, including the feedstock measurements, were performed all together, after the tests. Once the concentration of oxygen in the feedstock salt was calculated, it was found that the stoichiometric amount of Mg required to purify the salt was around 0.01 wt %. This means even when 0.05 wt % Mg metal was used it represented a 5-fold excess. The IGFA measurements, including the feedstock measurements, were performed all together, after the tests.

The effect of processing time (length of time at 670 °C) is shown in Figure 9. The baseline processing time of 3h was chosen to replicate the NREL process. The effect of processing time on salt oxygen concentration was examined by holding the salt at a shorter time of 1h or a longer time of 6h at 670 °C.

**Figure 9:**
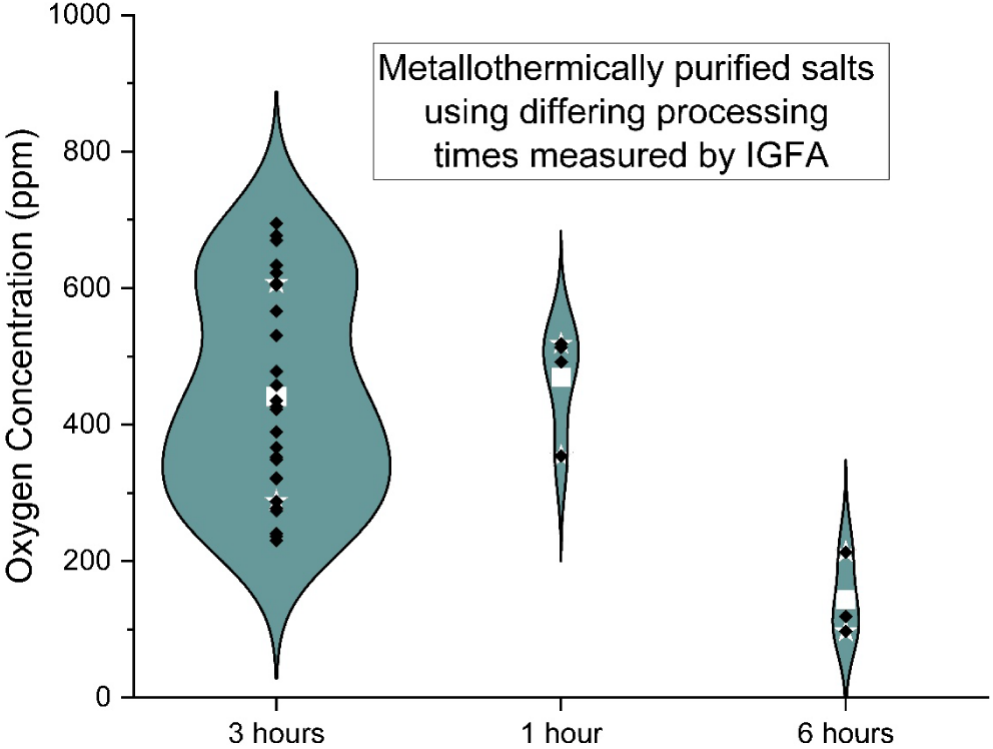
Oxygen concentration for metallothermically purified salt with varying processing times (time at 670 °C).

Reducing the processing time did not have a statistically significant effect in these tests. Increasing the processing time reduced the oxygen content significantly, with a confidence greater than 99%. The average oxygen concentration decreased from 442 ppm for the baseline tests to 143 ppm for the test run for 6 h. As discussed in a previous section, and by Zhao, it is likely that sparging rate, predrying, and processing time are all interrelated. An important consideration when discussing this process is settling. Metallothermic purification relies on the impurities settling into a slag layer. Filtration has been shown to be minimally effective as the particle size of many of the precipitates may be <1 μm. Therefore, increasing processing time to drive the reaction further toward completion may be more efficacious than increasing the sparging rate. The density of the salt at 670 °C, 1.6 g/cm^3^,^17^ is much lower than the density of MgO, 3.6 g/cm^3^.^31^ This means, depending on particle size, the MgO should settle out. However, the density of the Mg metal is close to that of the salt. It is around 1.6 g/cm^3^ when it is molten, but increases to just under 1.7 g/cm^3^ when it solidifies. More tests are needed to determine how settling, sparging, and temperature interact to produce a consolidated slag.

## Conclusions

Metallothermic purification of chloride salts is an attractive technique to improve the quality of off-the-shelf salts because it is relatively simple and eliminates the need to handle corrosive purification gases like Cl_2_ or HCl. This work tested the effects of changing operating parameters for metallothermic purification of chloride salt on the oxygen content in the resulting product, measured via IGFA. Processing parameters that were considered included: sparge gas flow rate, flush gas flow rate, amount of magnesium added, and processing time.

As-received salt contained 3466 ppm of oxygen. Melting alone decreased oxygen concentration to 889 ppm. The standard/baseline purification procedure used a sparge gas flow rate of 4.7 cm^3^/s, a flush gas flow rate of 16.5 cm^3^/s, 0.25 wt % of Mg added, and 3 h processing time at 670 °C. Performing the purification with these parameters further reduced the oxygen content to 442 ppm.

Altering the sparge gas and flush gas flow rates was found to have no discernible effect on the purification. Additional tests are needed to assess whether this behavior will persist at larger scales and with feedstocks containing higher concentrations of impurities.

Altering the mass of Mg added to the salt had no significant effect on the purity for the conditions tested within this work. It should be clarified that the masses of Mg that were tested all exceeded the stoichiometric amount required to reduce the oxygen in the feedstock salt used in this work.

### The Parameter That Had the Most Significant Effect Was the Processing Time

Reducing the processing time from 3 to 1h had little effect, but increasing the processing time from 3 to 6h reduced the amount of oxygen in the salt from 442 to 143 ppm.

The statistical significance of results were assessed using *t* tests (comparison of means) at a confidence level of >99%. An uncertainty analysis (Appendix A) showed that the theoretical measurement uncertainty that can be achieved with this machine and the standards that were used in this study is around 9%.

## Figures and Tables

**A1 figA1:**
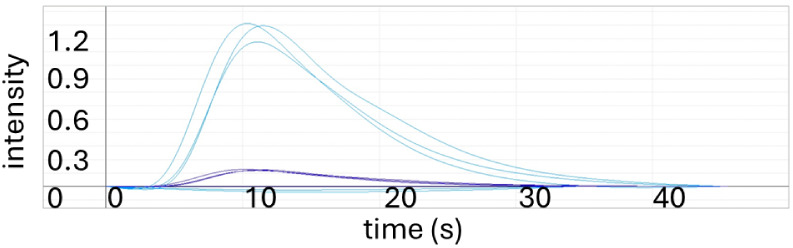
Measurement curves from the samples used to build Calibration 12 are shown. The graph shows the detector output, the area under the curve is the measured parameter. The dark blue line signifies measurements taken with the high concentration oxygen detector, and the light blue line signifies measurements taken with the low concentration oxygen detector.

**A2 figA2:**
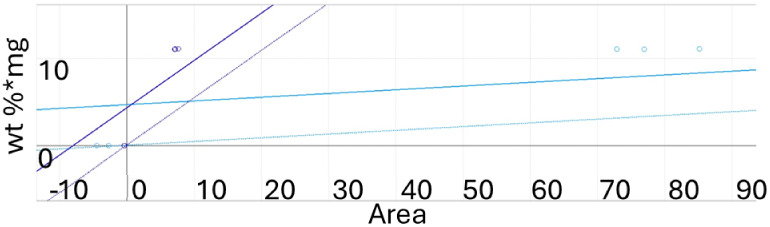
Calibration curves from Calibration 12 are shown. The graph shows the regression curves, as output by the detector. The dark blue line signifies measurements taken with the high concentration oxygen detector, and the light blue line signifies measurements taken with the low concentration oxygen detector.

**A3 figA3:**
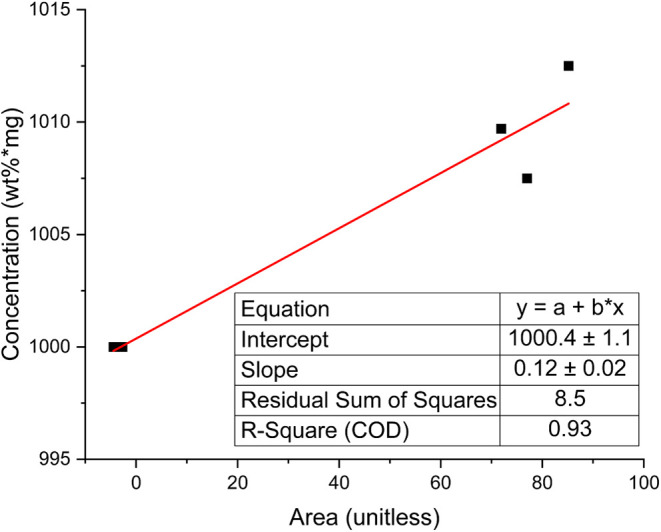
Calibration data from Calibration 12 is replotted for clarity.

## Appendix A: IFGA Uncertainty Analysis

For experimental investigations, quantifying uncertainty is crucial. The uncertainty of a group of measurements can generally be captured by considering two main sources of error: error related to the ability of the instrument(s) to measure the correct value (systematic), and variability in the data itself (statistical). Understanding the uncertainty well enables steps to be taken to minimize it and get better results in the end.

Statistical errors, or random errors, are a function of the samples themselves. The true variability of the samples cannot be changed without modifying the process, but some steps may be taken to reduce observed variability such as better sampling procedures, homogenization of the samples, and more uniform testing procedures. Systemic error is due to the inability of sensors to measure perfectly. In the combustion analyzer, for example, there is some limit of detection under which the IR sensor cannot confidently resolve oxygen, and some inherent uncertainty associated with the sensor itself. The sources of these statistical and systemic errors expected from the IGFA measurements are shown in Table [Table tbla1]

**A1 tbla1:** Expected Errors in IGFA for Measuring Oxygen in Salt Samples[Table-fn tbla1-fn1]

Measurement	Error	Typical value	Type	Instrument
Measured oxygen in sample $\left(O_{\mathrm{sample}}\right)$	propagated	23 to 1351 ppm avg. 465 ppm (*n* = 96)	Mixed	ELTRA
Area measured in sample $(A_{\mathrm{sample}}$	0.0024^+^ wt %·mg	47 ± 25 (*n* = 87) (calculated)	Systematic	ELTRA
Area measured in blank $\left(A_{\mathrm{blank}}\right)$	0.0024^+^ wt %·mg	-1.6 ± 1.6 (*n* = 27)	Systematic	ELTRA
Mass of sample $\left(m_{\mathrm{sample}}\right)$	42 g range-0.01 mg	N/A	Systematic	Balance (AND)
	210 g range-0.1 mg	139 ± 35 mg (*n* = 89)	Systematic	Balance (AND)
Transient calibration factor $\left(C_{\mathrm{cal}}\right)$	propagated	2.7 ± 0.7 (*n* = 12)	Random	N/A
Base calibration factor (factory)	0.0395	negligible	Random	N/A
Total mass $\left(m_{\mathrm{total}}\right)$	42 g range-0.01 mg	N/A	Systematic	Balance (AND)
	210 g range-0.1 mg	100 mg	Systematic	Balance (AND)
Mass of capsule $\left(m_{\mathrm{capsule}}\right)$	42 g range-0.01 mg	N/A	Systematic	Balance (AND)
	210 g range-0.1 mg	210 ±2 mg (*n* = 63)	Systematic	Balance (AND)
Mass of calibration standard $\left(m_{\mathrm{cal}}\right)$	0.1 mg	1009 ± 5 mg (*n* = 41)	random	Balance (Sartorius)
Oxygen in calibration standard $\left(O_{\mathrm{cal}}\right)$	0.0011 wt %*mg	0.011 wt %*mg	Random	N/A
Area measured in calibration standard $\left(A_{\mathrm{cal}}\right)$	0.0024^+^ wt %·mg	93 ± 19 avg. 70 (*n* = 41)	random	ELTRA

a +: The precision of the NDIR sensor is stated as 0.02 ppm, or 0.2% RSD in ELTRA’s specifications. This corresponds to 0.000002 wt %·mg. Assuming a sampling rate of 10 Hz and a sampling time of 120 s, this works out to 0.0024 wt %·mg·Hz·s, which cancels to 0.0024 wt %·mg.

These errors can be combined by the standard error propagation formula (eq [Disp-formula eqA1]).

A1
$$\mathrm{error}=\sqrt{{(\frac{\partial f}{\partial x})}^{2}\times s_{x}^{2}+{(\frac{\partial f}{\partial y})}^{2}\times s_{y}^{2}}$$

To propagate error, a function describing the dependence of the oxygen content on some variables must be constructed. To measure the amount of oxygen in the sample the area under the curve of intensity vs time for the IR detector is used, normalized by mass The amount of oxygen measured in the calibration is then subtracted out to give the final oxygen concentration (eq [Disp-formula eqA2]). The concentration of the calibration standards is 0.011 wt %, but because the mass of the steel pins varies the mass must be included. So the units used by the manufacturer are wt %*mg. The area under the curve is considered to be unitless.

The manufacturer suggests that a single point calibration be used and has a way to do the calibration integrated in their software. The measurement curves from the points used in Calibration 12 are shown in Figure [Fig figA1], as displayed on the machine. The calibration curves from Calibration 12 are shown in Figure [Fig figA2], as displayed on the machine. Finally, the data from the calibration curve is replotted in Figure [Fig figA3] to make it clearer. It should be mentioned that the calibration data from Calibration 12 represents a small fraction of the total calibration data collected for this uncertainty analysis.

The following equation (eq [Disp-formula eqA2]), given by the instrument manufacturer ELTRA via email correspondence, is used to describe the conversion from the measured area to the amount of oxygen in the sample (wt %). It is assumed that these variables are independent of one another.

$$O_{\mathrm{sample}}=\left(\frac{A_{\mathrm{sample}}-A_{\mathrm{blank}}}{m_{\mathrm{sample}}}\right)\times C_{\mathrm{cal}}\times C_{\mathrm{cal}}^{\mathrm{base}}$$
A2

where *O*_sample_, *A*_sample_, *A*_blank_, *m*_sample_, *C*_cal_, and C_cal_^base^ are the oxygen in the sample, the area measured from the sample, the area measured during calibration for the blank, the sample mass the measured calibration factor, and the base calibration factor, respectively. The base calibration factor does not change and is measured at the factory. For simplicity the error in this factor is assumed to be negligible. The partial derivatives of the independent variables are taken (eqs [Disp-formula eqA3]–[Disp-formula eqA6]) to build an error propagation equation (eq [Disp-formula eqA7]).

A3
$$\frac{\partial O_{\mathrm{sample}}}{\partial A_{\mathrm{sample}}}=\frac{C_{\mathrm{cal}}\times C_{\mathrm{cal}}^{\mathrm{base}}}{m_{\mathrm{sample}}}$$

A4
$$\frac{\partial O_{\mathrm{sample}}}{\partial A_{\mathrm{blank}}}=\frac{{-C}_{\mathrm{cal}}\times C_{\mathrm{cal}}^{\mathrm{base}}}{m_{\mathrm{sample}}}$$

A5
$$\frac{\partial O_{\mathrm{sample}}}{\partial C_{\mathrm{cal}}}=\frac{(A_{\mathrm{sample}}-A_{\mathrm{blank}})\times C_{\mathrm{cal}}^{\mathrm{base}}}{m_{\mathrm{sample}}}$$

A6
$$\frac{\partial O_{\mathrm{sample}}}{\partial m_{\mathrm{sample}}}=\frac{-(A_{\mathrm{sample}}-A_{\mathrm{blank}})\times C_{\mathrm{cal}}\times C_{\mathrm{cal}}^{\mathrm{base}}}{m_{\mathrm{sample}}^{2}}$$

A7
$$s_{O_{\mathrm{sample}}}=\sqrt{\begin{array}{c} {(\frac{\partial O_{\mathrm{sample}}}{\partial A_{\mathrm{sample}}})}^{2}\times s_{IR}^{2}+{(\frac{\partial O_{\mathrm{sample}}}{\partial A_{\mathrm{blank}}})}^{2}\times s_{IR}^{2}\\ +{(\frac{\partial O_{\mathrm{sample}}}{\partial C_{\mathrm{cal}}})}^{2}\times s_{C_{\mathrm{cal}}}^{2}+{(\frac{\partial O_{\mathrm{sample}}}{\partial m_{\mathrm{sample}}})}^{2}\times s_{m_{\mathrm{sample}}}^{2} \end{array}}$$

To calculate the error of the calibration factor and sample mass, additional error propagations must be performed. The error of the mass can be calculated using the uncertainty in the scale for both the capsule and the salt (eq [Disp-formula eqA8]).

A8
$$m_{\mathrm{sample}}=m_{\mathrm{total}}-m_{\mathrm{capsule}}$$

where *m*_sample_, *m*_total_, and *m*_capsule_ are the mass of the salt sample, the total sample mass, and the mass of the capsule, respectively. The partial derivatives of the independent variables are taken (eqs [Disp-formula eqA9] and [Disp-formula eqA10]) to build an error propagation equation (eq [Disp-formula eqA11]). The error in the measured mass (eq [Disp-formula eqA12]) is a function of the balance uncertainty (0.1 mg).

A9
$$\frac{\partial m_{\mathrm{sample}}}{\partial m_{\mathrm{total}}}=1$$

$$\frac{\partial m_{\mathrm{sample}}}{\partial m_{\mathrm{capsule}}}=-1$$
A10

A11
$$s_{m_{\mathrm{sample}}}=\sqrt{{(\frac{\partial m_{\mathrm{sample}}}{\partial m_{\mathrm{total}}})}^{2}\times s_{\mathrm{balance}}^{2}+{(\frac{\partial m_{\mathrm{sample}}}{\partial m_{\mathrm{capsule}}})}^{2}\times s_{\mathrm{balance}}^{2}}$$

A12
$$s_{m_{\mathrm{sample}}}=\sqrt{2\times {\left(1\right)}^{2}\times 0.1^{2}}=1.14\;\mathrm{mg}$$

The error in the calibration is a function of the balance uncertainty (0.1 mg), calibration standards (0.0011 wt %·mg), and the detector itself (0.0024 wt %·mg). To calibrate the machine a series of standards are run and the results are linearly regressed in a single point calibration (eq [Disp-formula eqA13]). In addition, blanks are run.

$$C_{\mathrm{cal}}=\frac{m_{\mathrm{cal}}\times O_{\mathrm{cal}}}{A_{\mathrm{cal}}\times C_{\mathrm{cal}}^{\mathrm{base}}}$$
A13

where *m*_cal_, *O*_cal_, and *A*_cal_ are the mass of the calibration standard, the concentration of the oxygen standard, and the area measured from the calibration, respectively. The partial derivatives of the independent variables are taken (eqs [Disp-formula eqA14]–[Disp-formula eqA16]) in order to build an error propagation equation (eq [Disp-formula eqA17]).

A14
$$\frac{\partial C_{\mathrm{cal}}}{\partial m_{\mathrm{cal}}}=\frac{O_{\mathrm{cal}}}{A_{\mathrm{cal}}\times C_{\mathrm{cal}}^{\mathrm{base}}}$$

A15
$$\frac{\partial C_{\mathrm{cal}}}{\partial O_{\mathrm{cal}}}=\frac{m_{\mathrm{cal}}}{A_{\mathrm{cal}}\times C_{\mathrm{cal}}^{\mathrm{base}}}$$

A16
$$\frac{\partial C_{\mathrm{cal}}}{\partial A_{\mathrm{cal}}}=\frac{{-m}_{\mathrm{cal}}\times O_{\mathrm{cal}}}{A_{\mathrm{cal}}^{2}\times C_{\mathrm{cal}}^{\mathrm{base}}}$$

A17
$$s_{C_{\mathrm{cal}}}=\sqrt{{(\frac{\partial C_{\mathrm{cal}}}{\partial m_{\mathrm{cal}}})}^{2}\times s_{\mathrm{balance}}^{2}+{(\frac{\partial C_{\mathrm{cal}}}{\partial O_{\mathrm{cal}}})}^{2}\times s_{\mathrm{standard}}^{2}+{(\frac{\partial C_{\mathrm{cal}}}{\partial A_{\mathrm{cal}}})}^{2}\times s_{IR}^{2}}$$

A18
$$s_{C_{\mathrm{cal}}}=\sqrt{\begin{array}{c} {\left(\frac{0.011}{70\times 0.0395}\right)}^{2}\times 0.1^{2}+{\left(\frac{1000}{70\times 0.0395}\right)}^{2}\\ \times 0.0011^{2}+{\left(\frac{-1\times 0.011}{70^{2}\times 0.0395}\right)}^{2}\times 0.02^{2} \end{array}}=0.30$$

With these numbers calculated (eqs [Disp-formula eqA12] and [Disp-formula eqA18]), they can be plugged into the original error propagation formula (eq [Disp-formula eqA7]) for an average concentration of 465 ppm O.

A19
$$s_{O_{\mathrm{sample}}}=\sqrt{\begin{array}{c} {\left(\frac{2.7\times 0.0395}{100}\right)}^{2}\times 0.0024^{2}+{\left(\frac{-2.7\times 0.0395}{100}\right)}^{2}\times 0.0024^{2}\\ +{\left(\frac{(42+1.6)\times 0.0395}{100}\right)}^{2}\times 0.30^{2}+{\left(\frac{(42+1.6)\times 2.7\times 0.0395}{100^{2}}\right)}^{2}\times 0.14^{2} \end{array}}=0.00417$$

The final error (eq [Disp-formula eqA19]) is 41.7 ppm. While the absolute number changes as a function of the amount of oxygen in the sample the percentage remains the same, about 9.0%. This is dominated by the error in the calibration standard, which can be traced back to eqs [Disp-formula eqA15] and [Disp-formula eqA17]. Reducing the uncertainty of the calibration standards by a factor of ten, from 11 ppm to 1.1 ppm, correspondingly reduces the error in the result by a factor of ten. Other sources of error (besides the calibration standards) account for a combined uncertainty of less than 0.1%. Therefore, minimizing the uncertainty in the calibration standards would significantly reduce the overall uncertainty.

## Nomenclature and Acronyms

**Notation**
*A*_blank_: area measured for the blank during calibration (unitless)
*A*_cal_: area measured for the standard during calibration (unitless)
*A*_sample_: area measured from the sample (unitless)
*C*_cal_: calibration factor (wt %*mg)
$C_{cal}^{base}$: calibration factor (unitless)
*M*: some active metal (Mg, Ca, Na, etc.)
*m*_cal_: calibration standard mass (mg)
*m*_capsule_: capsule mass (mg)
*m*_sample_: sample mass (mg)
*m*_total_: total mass (mg)
*O*_cal_: calibration standard oxygen concentration (wt %)
*O*_sample_: oxygen in the sample (wt %)
*s*_*x*_: error in variable *x*
$\frac{\partial f}{\partial x}$: partial derivative of function *f* with respect to variable *x*
**Acronyms**
ASTM: American Society for Testing and Materials
CSP: Concentrated Solar Power
CV: Cyclic Voltammetry
DOE: United States Department of Energy
DSC: Differential Scanning Calorimetry
ICP-MS: Inductively-Coupled Plasma Mass Spectrometry
IGFA: Inert Gas Fusion Analysis
IR: Infrared
KF titration: Karl Fisher titration
LOD: Level Of Detection
LIBS: Laser-Induced Breakdown Spectroscopy
MSR: Molten Salt nuclear Reactors
MSRE: Molten Salt Reactor Experiment
NDIR: Non-Dispersive InfraRed
NPV: Normal Pulse Voltammetry
NREL: National Renewable Energy Laboratory
OB: Optical Basicity
OCP: Open Circuit Potential
O/H impurities: Oxygen and Hydrogen based impurities
PTFE: PolyTetraFluoroEthylene
RSD: Relative Standard Deviation
SWV: Square Wave Voltammetry
TES: Thermal Energy Storage
TGA: ThermoGravimetric Analysis
UHP: Ultra High Purity
XRD: X-ray Diffraction
**Units**
cm: centimeter (10^–2^ m)
g: gram
hr: hour (3.6 × 10^3^ s)
in: inches (2.54 × 10^–2^ m)
K: degrees Kelvin
mg: milligram (10^–3^ g)
ml: milliliter (10^–3^ l)
mm: millimeter (10^–3^ m)
mW: milliwatt (10^–3^ W)
ppm: parts per million
s: second
SCFM: Standard Cubic Feet per Minute (∼4.72 × 10^2^ cm^3^/s)
W: watt
wt %: percent by weight
μm: micrometer (10^–6^ m)
%: percent
°C: degrees Celsius
